# The Genetic and Environmental Determinants of the Association Between Brain Abnormalities and Schizophrenia: The Schizophrenia Twins and Relatives Consortium

**DOI:** 10.1016/j.biopsych.2012.01.010

**Published:** 2012-05-15

**Authors:** Neeltje E.M. van Haren, Fruhling Rijsdijk, Hugo G. Schnack, Marco M. Picchioni, Timothea Toulopoulou, Matthias Weisbrod, Heinrich Sauer, Theo G. van Erp, Tyrone D. Cannon, Matti O. Huttunen, Dorret I. Boomsma, Hilleke E. Hulshoff Pol, Robin M. Murray, Rene S. Kahn

**Affiliations:** a University Medical Center Utrecht, Department of Psychiatry, Division of Neuroscience, Rudolf Magnus Institute, Utrecht, The Netherlands; b VU University Amsterdam, Netherlands Twin Register, Department of Biological Psychology, Amsterdam, The Netherlands; c Institute of Psychiatry, Kings College London, London, United Kingdom; d St. Andrew's Academic Centre, King's College London, Institute of Psychiatry, Northampton, United Kingdom; e Department of General Adult Psychiatry, Centre for Psychosocial Medicine, Heidelberg, and SRH Klinikum, Karlsbad-Langensteinbach, Germany; f University of Jena, Department of Psychiatry, Germany; g Department of Psychiatry and Human Behavior, University of California Irvine, Irvine, California; h Departments of Psychology and Psychiatry and Biobehavioral Sciences, University of California at Los Angeles, Los Angeles, California; i Department of Mental Health and Alcohol Research, National Health Institute, Helsinki, Finland

**Keywords:** Brain volumes, multicenter, phenotypic correlation, schizophrenia, sMRI, twins

## Abstract

**Background:**

Structural brain abnormalities are consistently found in schizophrenia (Sz) and have been associated with the familial risk for the disorder. We aim to define the relative contributions of genetic and nongenetic factors to the association between structural brain abnormalities and Sz in a uniquely powered cohort (Schizophrenia Twins and Relatives consortium).

**Methods:**

An international multicenter magnetic resonance imaging collaboration was set up to pool magnetic resonance imaging scans from twin pairs in Utrecht (The Netherlands), Helsinki (Finland), London (United Kingdom), and Jena (Germany). A sample of 684 subjects took part, consisting of monozygotic twins (*n* = 410, with 51 patients from concordant and 52 from discordant pairs) and dizygotic twins (*n* = 274, with 39 patients from discordant pairs). The additive genetic, common, and unique environmental contributions to the association between brain volumes and risk for Sz were estimated by structural equation modeling.

**Results:**

The heritabilities of most brain volumes were significant and ranged between 52% (temporal cortical gray matter) and 76% (cerebrum). Heritability of cerebral gray matter did not reach significance (34%). Significant phenotypic correlations were found between Sz and reduced volumes of the cerebrum (−.22 [−.30/−.14]) and white matter (−.17 [−.25/−.09]) and increased volume of the third ventricle (.18 [.08/.28]). These were predominantly due to overlapping genetic effects (77%, 94%, and 83%, respectively).

**Conclusions:**

Some of the genes that transmit the risk for Sz also influence cerebral (white matter) volume.

Although the causes of schizophrenia (Sz) are not completely understood, the importance of genetic factors has been firmly established by family, twin, and adoption studies. There is evidence for a substantial genetic contribution to the etiology of Sz, with heritability estimates up to 85% (1,2). Over the last decade a large number of linkage and (genome-wide) association studies have been carried out (3–5). The genes thus far identified only explain a small part of the genetic risk for Sz, although many seem to be involved in neurodevelopmental processes (6–8). This suggests that some of the genetic risk leading to the development of Sz is expressed as abnormal brain development.

In fact, abnormal brain structure is one of the most robust biological features of Sz (9). Volume loss in the prefrontal lobes, thalamus, superior temporal cortex, and hippocampus have consistently been demonstrated with in vivo and postmortem approaches (10). It is well-known that brain volume (BV) is highly heritable (11,12) and that the brain abnormalities found in Sz cosegregate with the illness within families (13,14). Furthermore, twin probands have smaller whole BVs than their nonaffected co-twins, who in turn have smaller brains than healthy twins (15–17). Hulshoff Pol *et al*. (18) showed that smaller white matter volume reflects the expression of genetic risk, whereas less gray matter is related to environmental risk factors. Thus, family and twin studies to date suggest that some of the brain abnormalities in Sz can be attributed to the genes conferring risk for this disorder. Although several twin-studies have tried to quantify the relative contribution of genetic and nongenetic factors to these brain abnormalities, these studies have all been small (due to recruitment challenges) and lacked power, leading to unreliable estimates. Furthermore, for a phenotype or endophenotype to be useful in the search for disease-related genes, it should not only be heritable but also share genetic variance with the risk for the disorder. Few studies have tested for such pleiotropic effects. The STAR (Schizophrenia Twins and Relatives) consortium was established to address these methodological problems, combining brain imaging data from most of the available twin samples with Sz.

Although the need to pool twin samples to gain sufficient statistical power is not disputed, it is a great challenge to combine structural magnetic resonance imaging (MRI) data, given the variability of magnetic resonance scanners and acquisition protocols between sites. The ADNI (Alzheimer's Disease Neuroimaging Initiative) (19) and BIRN (Biomedical Informatics Research Network) studies (20,21) developed useful recommendations on how best to conduct multicenter imaging protocols; however, in the present multicenter study, tuning of the MRI acquisition protocols was not possible because scans of the twin pairs were already acquired. Therefore, a group of calibration subjects was scanned at all participating research sites, with the same acquisition protocols the twins were scanned with. The image processing pipeline algorithm was optimized by tuning two calibration factors that separated gray matter, white matter, and cerebrospinal fluid. This resulted in comparable between-site volumes for whole brain, cerebral gray and white matter volume, cerebellum, and third and lateral ventricles across most sites (22) (see also Methods and Materials). Consequently, we are confident that using the calibration factors from this study in the processing of the MRI twin data resulted in a reliable and unique powered sample. We investigated the relative contribution of genetic and environmental influences on the association between Sz liability and BV.

## Methods and Materials

### Subjects

The STAR consortium pooled all twin samples with MRI brain scans, collected at the Institute of Psychiatry, London (United Kingdom), University of Helsinki, Helsinki (Finland, in collaboration with University of California, Los Angeles), University of Jena, Jena (Germany), Universitätskliniek Heidelberg, Heidelberg (Germany), and the University Medical Center Utrecht, Utrecht (UMCU, The Netherlands), to increase power for variance component analyses. All available MRI scans were collated within the Department of Psychiatry at the UMCU and processed with our processing pipeline (see following). Previously a calibration and data compatibility study concluded that the Heidelberg scans could not be reliably pooled with the other sites, due to low interscanner reliability (intraclass coefficients) of the gray and white matter volumes (22).

High-quality MRI scans were available for 684 individuals, including 142 patients with Sz and 542 unaffected individuals (co-twins and control twins). Mean age in the total sample was 37.97 years (SD = 11.31). Table 1 lists the numbers of included individuals as a function of disease state, site, age, and gender.

Information on recruitment and psychiatric assessment for each site is described in Supplement 1.

### MRI Processing

Scans were acquired on Philips (Utrecht, The Netherlands; Jena, Germany), GE (London, United Kingdom), and Siemens (Munich, Germany) systems. Differences in scan acquisition between sites concerned, among others, scan orientation (coronal or sagittal) and voxel dimensions (see Table S1 in Supplement 1). The London twins were scanned at St. Georges Hospital (*n* = 78) and at the Maudsley hospital (*n* = 54) on identical 1.5-T GE Signa scanners with slightly different acquisition protocols (16). Six twins were scanned on both scanners. Intraclass correlation coefficient (ICC) estimates ranged from .84 (cerebral gray matter) to .95 (lateral ventricles).

Image processing of the brain scans from Utrecht, London, and Jena was done on the neuroimaging computer network of the Department of Psychiatry in Utrecht. The reproducibility of the segmentation process on scans from the Utrecht scanner was established with ICC (23) and were .96 or higher for all structures (24–26). The T1-weighted images were first put into Talairach orientation (no scaling) (27). If a T2-weighted image was available (Utrecht and London), an intracranial volume was automatically segmented from this image. After registration to the T1-weighted image with a mutual information maximization algorithm (28), this segment served as a mask for further segmentation steps. If no T2-weighted image was available, an intracranial mask was manually segmented from the T1-weighted image.

The T1-weighted images were corrected for scanner radiofrequency-field nonuniformity (29). Total brain segmentations were done automatically, with mathematical morphology operations, on the basis of thresholds obtained from the steepest slope of the gray matter peak in intensity histograms of the intracranial region (i.e., the cerebrospinal fluid/gray matter separation threshold is the position of the steepest slope multiplied by a calibrated factor [.73 for scans from all sites]). This has been validated before (22,25).

Cerebellum and lateral and third ventricular segmentations were carried out semi-automatically on the basis of histogram analyses followed by mathematical morphology operations on the T1-weighted image. Anatomic knowledge-based selection principles were used for these segmentations. All segments were checked and manually corrected if necessary.

Separation of cerebral gray and white matter was done by applying a single threshold to the voxels of the total brain in the T1-weighted image. For each image, the threshold was obtained automatically from the T1-weighted intensity histogram (25). We have previously shown (25) that a scaling factor has to be calibrated, because of the dependence of the shapes of the gray and white matter distributions on the acquisition parameters. It was calculated for Utrecht scans on the basis of a comparison in 80 scans, where the gray/white separation had been determined manually twice by three raters (*f*_*gw*_ = .960). For scans from London and Jena, the threshold factors for gray/white separation were optimized (22) (London: *f*_gw_ = .980; Jena: *f*_gw_ = .970).

To obtain the cerebral white matter segmentation of the image, a selection of all voxels in the cerebrum with intensities above the calibrated threshold was made. The gray matter segment was calculated from the difference between the cerebrum segment and cerebral white matter segment. Absolute volumes were estimated from the product of the number of voxels in each segment and the voxel volume.

In addition, frontal, parietal, temporal, and occipital lobes were manually demarcated on a model brain (30) that was selected earlier among 200 brain images of subjects between 16 and 70 years of age (31). Brain images were registered to the model brain through the Automatic Non-linear Image Matching and Anatomical Labeling (ANIMAL) algorithm (32) to remove global differences in size and shape of the individual brains. The inverse of the transformation process registered the manual segmentations of the model brain to the brain images of all subjects. The gray matter segments from the individual brain images were used to identify cortical gray matter for each individual lobe.

The Helsinki images were processed with a processing pipeline at the University of California at Los Angeles. In brief, a semi-automated method based on Montreal Neurological Institute tools was used to create brain-only masks, and trained operators subsequently outlined the brain parenchyma on each section, eliminating pixels corresponding to the skull and meninges. A radiofrequency bias field correction algorithm eliminated intensity drifts due to scanner field inhomogeneity (29). The Functional MRI of the Brain's Automated Segmentation Tool (FAST) (33) was used to classify tissue types into gray matter, white matter, and cerebrospinal fluid.

Because a calibration dataset was available from four subjects scanned in Helsinki and Utrecht (i.e., eight scans in total), we were able to estimate the reliability of pooling data from the two processing procedures. All eight scans were processed with both procedures, and ICCs were measured for cerebral (gray and white matter) volumes. These were all >.99 after including site as a covariate. Therefore, cerebral (gray and white matter) volumes from the Helsinki twin sample were included in the analysis.

### Statistical Analysis

#### Polychoric Correlations

To analyze the Sz data, a liability threshold model was applied, which assumes that disease risk is distributed normally (with a variance of one and mean of zero) (34). A threshold separates the distribution into affected and unaffected individuals. The liability distribution is unobserved, and its variance can be attributed to genetic and nongenetic factors. Resemblance between twins on the liability distribution is summarized with tetrachoric (dichotomous traits) or polychoric correlations (traits with > 1 threshold). The twin data were analyzed as a function of zygosity (monozygotic [MZ] and dizygotic [DZ]) with Sz status (yes/no) and BVs as outcome variables. Brain volumes were categorized into five classes (with roughly equal numbers/class) after regressing out the effects of one site (i.e., Helsinki, because of the different processing procedure), gender, age, and intracranial volume.

Polychoric (twin) correlations between the underlying liabilities for Sz and BV were estimated as a function of zygosity. The correlations estimated are: cross-trait within-member (Sz_m1_–BV_m1_), within-trait cross-members in pairs of MZ twins or DZ twins (Sz_m1_–Sz_m2_ or BV_m1_–BV_m2_), cross-trait cross-member in pairs of MZ twins or DZ twins (Sz_m1_–BV_m2_ or BV_m1_–Sz_m2_). Several constraints were imposed. The cross-trait within-member correlations were constrained to be equal across all individuals in the sample, yielding only one Sz–BV correlation. The cross-trait cross-member correlations were constrained to be equal within the MZ twin and DZ twin group separately, such that Sz_m1_–BV_m2_ = Sz_m2_–BV_m1_ (Table 2). The thresholds for BVs were constrained to be equal across groups.

#### Interpretation of Correlations

Significant cross-trait within-member correlations might imply common etiological influences. The power to distinguish between different factors (e.g., genetic or environmental) causing the correlation is derived from the MZ and DZ cross-trait cross-member correlations of the MZ twin pairs and DZ twin pairs. Significant cross-trait cross-member correlations (e.g., Sz_m1_–BV_m2_) imply that these common etiological influences are familial. Whether these familial influences are genetic or environmental in origin is indicated by the MZ/DZ ratio of these correlations. A 2:1 ratio is indicative of additive genetic effects, whereas a 1:1 ratio suggests the influences of (shared) common environment in inducing a correlation between the traits. Nonsignificant cross-trait cross-member correlations imply that the etiological influences on Sz and BV are due to specific unique environmental effects (for background information see Neale *et al.* [35]).

#### Genetic Model Fitting

**Figure 1:**
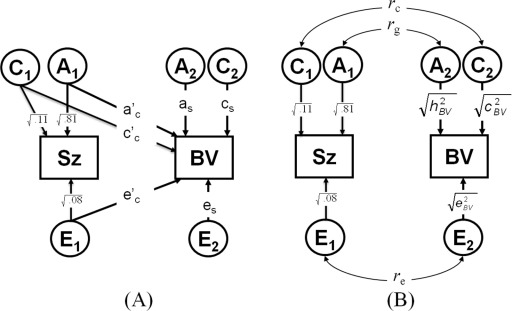
**(A)** The applied bivariate model. Additive genetic effects: A; common environmental: C; and unique environmental effects: E are specified such that factors A_1_, C_1_, and E_1_ influence both schizophrenia (Sz; path a_c_, c_c_, e_c_) and brain volume (BV; path a'_c_, c'_c_, e'_c_), inducing a familial covariance that is either genetic (a_c_* a'_c_) or environmental (c_c_ * c'_c_) and an individual specific environmental covariance (e_c_ * e'_c_). Factors A_2_, C_2_, and E_2_ are specific to BV (a_s_, c_s_, e_s_). **(B)** The standardized correlated-factors solution, where the paths are the square roots of the heritabilities (h^2^), c^2^, and e^2^ and the double-headed arrows are the genetic c and e correlations (*r*_g_, *r*_c,_*r*_e)_. The proportion of the phenotypic correlation (*r*_ph_) due to A effects can then be calculated by (√ *h*^2^_SZ_ * *r*_g_ * √ *h*^2^_BV_), the part due to C effects by (√ *c*^2^_SZ_ * *r*_c_ * √ *c*^2^_BV_), and the part due to E effects by (√ *e*^2^_SZ_ * *r*_e_ * √ *e*^2^_BV_). For ascertainment correction, the fixed model parameters for schizophrenia are: *h*^2^ = .81, *c*^2^ = .11, *e*^2^ = .08.

The aim of this study is to examine the extent of the correlation between Sz and BVs due to genetic overlap or to common environmental effects. Mx Statistical Modeling (36) was used for maximum likelihood genetic model fitting to directly estimate model parameters from the observed data. The genetic bivariate model is illustrated in Figure 1A. Additive genetic effects (A), common environmental (C) and unique environmental effects—including measurement error (E)—are specified such that factors A_1_, C_1_, and E_1_ influence both Sz (path a_c_, c_c_, e_c_) and BV (path a‘_c_, c‘_c_, e‘_c_), inducing a familial covariance that is either genetic (a_c_* a'_c_) or environmental (c_c_ * c'_c_) and an individual specific environmental covariance (e_c_ * e'_c_). Factors A_2_, C_2_, and E_2_ are specific to BV (a_s_, c_s_, e_s_). If there is no relevance in the specific order of the variables, the solution of this Cholesky decomposition can be standardized in a correlated-factors model (Figure 1B), where for example the paths from A_1_ to Sz and A_2_ to BV are the square roots of their heritabilities, and where the correlational path between A_1_ and A_2_ is the genetic correlation (*r*_g_). The part of the phenotypic correlation (*r*_ph_), due to genetic effects can then be calculated by (√ *h*^2^_SZ_ * *r*_g_ * √ *h*^2^_BV_), the part due to C by (√ *c*^2^_SZ_ * *r*_c_ * √ *c*^2^_BV_) and the part due to E by (√ *e*^2^_SZ_ * *r*_e_ * √ *e*^2^_BV_).

#### Ascertainment Correction

The data are from twin pairs selected on the basis of the presence or absence of Sz rather than from a random population sample, requiring a correction for ascertainment. We corrected for ascertainment by constraining the genetic model parameters for Sz to constant values: point estimates (h^2^ = .81, c^2^ = .11, e^2^ = .08) from a recent meta-analysis (37) and the liability threshold to give a prevalence consistent with a lifetime risk of 1%. In contrast, the model parameters for brain structure (including liability thresholds) as well as the relationship with Sz are free parameters to be estimated from the data (Figure 1). This correction in the polychoric correlation scripts entails fixing the MZ and DZ/sib correlations for Sz to h^2^+c^2^ = .92 and to .5h^2^ + c^2^ = .515, respectively. This model has been described elsewhere in more detail (17) and applied to other Sz studies (38–40).

## Results

### Polychoric Correlations

**Figure 2:**
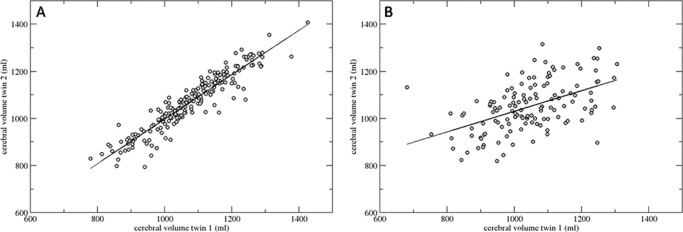
Correlation between cerebral volume of Twin 1 with Twin 2 for (**A)** all monozygotic (*n* = 196) and (**B)** all dizygotic (*n* = 131) twin pairs, irrespective of illness.

Correlations between the underlying liabilities for Sz and each BV are presented in Table 2. First, the ratios of the MZ and DZ twin correlation indicate a significant contribution of genetic effects to the observed variation for each of the BVs (Figure 2). Second, significant cross-trait within-member correlations (column 1) were present for: 1) reduced cerebral and cerebral white matter volume, and 2) increased third ventricle volume. The significant MZ and DZ cross-member cross-trait correlations for each volume do support a genetically mediated association with risk for Sz. This is formally tested in the genetic model-fitting analyses.

### Genetic Model Fitting

The results from the ACE model-fitting are presented in Table 3. Most of the BVs measured were significantly heritable and ranged between 52% (temporal cortical gray matter) and 76% (total cerebral volume). Heritability of cerebral gray matter (in particular, cortical frontal and occipital: 43% and 21%, respectively) did not reach significance (34%). Familial (common) environmental influences (c^2^) on the variation in each of the volumes were nonsignificant, except for cortical occipital gray matter volume (43%). The genetic (r_g_), shared-environmental (r_c_) and unique-environmental correlations (r_e_) indicate that the source of the significant phenotypic overlap between risk for Sz and cerebral volume decrease was due to genetic and unique-environmental factors (including correlated measurement error). For gray matter (in particular, frontal and temporal cortex) volume and lateral ventricle volume, these were all due to nonfamilial factors (i.e., correlated unique-environmental factors).

Table S2 in Supplement 1 shows the more parsimonious AE models, where all nonsignificant common-environmental effects are ignored. There was a significant genetic correlation between reduced cerebral volume and risk for Sz (rg = −.21, 95% confidence interval [CI]: −.31 to −.10). The phenotypic correlation between reduced cerebral volume and Sz risk of −.22 (r_ph_) could be broken down, with 77% due to common genetic factors (rph-a = −.17, 95% CI: −.25 to −.08), and 23% due to correlated subject-specific environmental factors (rph-e = −.05, 95% CI: −.08 to −.02).

Furthermore, for both reduced cerebral white matter and increased third ventricle volume, there was a significant phenotypic correlation with risk for Sz (r_ph_ = −.17, 95% CI: −.25 to −.09; r_ph_ = .18, 95% CI: .08 to .28, respectively). Genetic influences contribute significantly to the association between both structures and Sz liability. For cerebral white matter volume, overlapping A (genetic) factors = −.15 (95% CI: −.24 to −.07) accounting for approximately 94%. For increased third ventricle volume the part due to overlapping A factors was .15 (95% CI: .05 to .25) and an overlap of approximately 83%. For cerebral gray matter volume (in particular, cortical frontal and occipital) the influence of common environmental factors (C) could not be dropped from the model. A near-significant phenotypic correlation was found between gray matter and Sz (r_ph_ = −.08, 95% CI: −.16 to .00).

## Discussion

In the largest twin study to date of Sz and BVs (*n* = 684 individuals) we examined the relative contributions of genetic and environmental influences on the association of BVs and Sz. In an international multicenter collaboration we pooled data from four research centers, creating a uniquely powered twin cohort. We found a small but significant association between Sz and a smaller cerebral volume, of which 77% can be explained by genetic factors that influence both the lower cerebral volume and (the risk for developing) Sz. Lower cerebral white matter volume as well as larger third ventricular volume also showed a significant association with the liability for Sz, which was largely explained by genetic factors. These accounted for significant portions of the phenotypic correlations (94% and 83%, respectively), indicating that smaller cerebral (white) matter and larger third ventricle are related to the genetic risk to develop Sz. Thus, our results suggest that the smaller total cerebral volume and particularly that of white matter and larger third ventricle volume is linked to Sz, mainly through genetic factors that are associated to the disorder. In contrast, a significant environmental correlation for cerebral gray matter (−.36; 95% CI: −.55 to −.15) and cortical frontal gray matter (−.54; 95% CI: −.74 to −.29) was found, without a significant phenotypic association between smaller cerebral or cortical frontal and occipital gray matter volume and liability to Sz. Interestingly, there are contrasting contributions (although not significant) of genetic and common environmental factors. In other words, a negative association was found between genetic factors and lower volume, whereas common environmental factors showed a positive association with larger gray matter volume. This is in line with a recent study in MZ twins concordant and discordant for Sz showing a heterogeneous pattern of prefrontal volume reduction in twins with Sz. Medial and orbital frontal cortex showed significant volumetric reductions in twins with Sz only if they were concordant but not discordant for the disorder, relative to control subjects. In contrast, inferior frontal cortex showed no statistically significant differences across groups, whereas superior frontal cortex was reduced in Sz patients, regardless of their concordance status (41).

That impaired brain development is important in the pathogenesis of Sz is not new (42,43). Evidence from both genetic and epidemiological studies that aberrant neurodevelopment is crucial in the risk for Sz is mounting. Association studies have identified common risk variants that are associated with Sz (e.g., neuregulin, dysbindin, Disrupted in Schizophrenia 1 [DISC1]). In addition, small structural changes (copy number variants) in the genome seem causative of Sz. Interestingly, these genes as well as the copy number variants seem to influence neurodevelopment, cell signaling, and synaptic functions (44). Moreover, Fatemi and Folom (45) imply that Neuregulin-I and DISC1 not only play a key role in brain development but are likely to be functionally convergent.

Epidemiological data show that deficits and delays in cognitive development (46–48), emotional problems, interpersonal difficulties, and impairments in neuromotor and receptive language (48) are present during childhood and adolescence in individuals who will later go on to develop Sz. Crucially, the risk for Sz is increased in those with a lower IQ score, in particular in the presence of impaired nonverbal reasoning (49). Furthermore, recent work has shown that there are significant common genetic influences acting on the covariance between IQ, schizotypy, abnormal social functioning, and schizophrenia (50). This suggests that the same genetic factors that impair intelligence, increase schizotypy, impede social development, and decrease BV in childhood also determine the liability to Sz (39,50).

Although we found a significant association between Sz and smaller cerebral volume, the correlation is relatively low, r_ph_ = −.22. This subtle effect might not be surprising, because it was not expected that the risk for Sz explains a large part of the variance in cerebral volume. A correlation of −.22 indicates that susceptibility for Sz explains 4.8% of the variance, which is small but by no means negligible. This is even lower for white matter volume (−.17). Importantly, our results show that this effect in cerebrum and cerebral white matter is largely (77% and 94%, respectively) explained by shared genetic rather than environmental or disease-related factors. The genetic contribution to association between white matter volume and Sz liability has been suggested previously with a classic repeated measures General Linear Model analyses in the Utrecht discordant twin sample (51). Of note, these data are also part of the current pooled sample.

Our findings must be viewed in light of several methodological limitations. First, this pooled twin sample is not a population-based sample of twins with Sz and healthy control twins from the United Kingdom, The Netherlands, and Germany, except for the twin sample from Helsinki. Second, this study found no significant shared environment contribution to the variation in BVs, which rules it out as a possible source of overlap with the disorder. However, by constraining the BV familial environmental paths to zero in the AE model for some volumes, its effects (even if very small) will automatically be apportioned to the genetic component, therefore inflating the genetic correlation between BV and disorder. Third, we cannot distinguish how much each site contributes to the explained variation in BV due to A or E. Fourth, in the present study we cannot account for biological gene × environment interaction effects, because in the classical twin model the effects of interaction and correlation between latent A, C, and E factors is assumed to be zero. Twin data on their own cannot resolve these issues. There are (rather complex) designs that enable ways of testing these effects (52), but that is beyond the scope of most twin studies.

Finally, the findings of our study are limited to global BV measures. The advantage of global BVs is their robustness and stability in measurements, as is indicated by the relatively high ICCs we found across sites. In contrast, the reliability of voxel-based and cortical thickness measurements seem to differ between brain areas (53). We can make no anatomically specific inferences about the location of the focal gray and white matter deficits and how they might be differently related to the genetic and environmental risk to develop Sz. Future studies should deploy more focally sensitive approaches to brain anatomy with voxel-based morphometry or cortical thickness measurements. In addition, associations with other candidate endophenotypic markers such as intellectual functioning (IQ) need to be investigated.

In conclusion, we found that genetic factors largely explain the modest but significant association between Sz and total cerebral and white matter volume decrease and increase in third ventricle volume. These findings indicate that some of the genes that increase the risk to develop Sz are likely to be involved in crucial neurodevelopmental processes in the brain. Thus, evidence from genetic, epidemiological, and—as indicated by the current results—neuroimaging studies seem to converge on the fact that the risk to develop Sz is the consequence of genetically mediated aberrant neurodevelopment.

## Figures and Tables

**Table 1:** Number of Twins, Mean Age, and Gender Distribution of the Samples/Site

	Helsinki			Jena			London			Utrecht		
	*n*	Age (yrs)	Gender (F/M)	*n*	Age (yrs)	Gender (F/M)	*n*	Age (yrs)	Gender (F/M)	*n*	Age (yrs)	Gender (F/M)
MZ-Conc	13	42.31 (8.44)	6/7				38	35.26 (9.13)	9/29			
MZ-Disc Pt	14	47.93 (3.63)	8/6	11	34.46 (11.07)	6/5	14	30.71 (10.86)	4/10	13	37.52 (11.18)	6/7
Co-Twin	15	48.20 (5.36)	9/6	11	34.45 (11.07)	6/5	17	32.51 (12.92)	7/10	13	37.55 (11.15)	6/7
MZ-HC	48	47.77 (3.48)	22/26	16	34.98 (12.62)	8/8	53	36.43 (10.13)	20/33	134	33.99 (10.93)	66/68
DZ-Disc Pt	23	47.43 (5.03)	11/12				3	43.88 (17.24)	2/1	13	35.38 (11.04)	6/7
Co-Twin	23	48.30 (5.57)	12/11				3	43.88 (17.24)	2/1	13	35.23 (10.58)	6/7
DZ-HC	50	49.32 (4.68)	29/21				4	37.00 (13.86)	0/4	142	32.66 (10.35)	81/61

*N* = 684. Age given in years (SD). Conc, concordant; Disc, discordant; DZ, dizygotic; F, female; HC, healthy control subjects; M, male; MZ, monozygotic; Pt, patient.

**Table 2:** Polychoric Correlations Within-Member Cross-Trait, Cross-Member Within-Trait, and Cross-Member Cross-Trait for Sz and MRI BVs

BVs	Within-Member Cross-Trait (Sz_m1_– BV_m1_ = Sz_m2_ – BV_m2_) Whole Sample	Cross-Member Within-Trait (BV_m1_ – BV_m2_)		Cross-Member Cross-Trait (Sz_m1_ – BV_m2_ = Sz_m2_ – BV_m1_)	
		MZ Pairs	DZ Pairs	MZ Pairs	DZ Pairs
Cerebrum	−.22 (−.30 to −.14)^a^	.77 (.69 to .82)^a^	.25 (.04 to .44)^a^	−.17 (−.25 to −.09)^a^	−.09 (−.22 to .04)
Cerebral Gray	−.08 (−.11 to .00)	.57 (.45 to .66)^a^	.40 (.20 to .56)^a^	−.02 (−.10 to .07)	.07 (−.06 to .20)
Cerebral White	−.17 (−.25 to −.09)^a^	.73 (.65 to .80)^a^	.41 (.23 to .55)^a^	−.16 (−.24 to −.07)^a^	−.14 (−.26 to −.01)^a^
Lateral Ventricles	.10 (.00 to .10)	.73 (.63 to .80)^a^	.37 (.13 to .56)^a^	.04 (−.07 to .14)	.00 (−.19 to .17)
3rd Ventricle	.19 (.09 to .29)^a^	.75 (.67 to .82)^a^	.40 (.16 to .59)^a^	.16 (.05 to .26)^a^	.18 (.00 to .35)
Frontal Cortical Gray	−.03 (−.13 to .06)	.56 (.43 to .67)^a^	.34 (.10 to .54)^a^	.07 (−.03 to .17)	−.07 (−.12 to .25)
Temporal Cortical Gray	−.04 (−.14 to .06)	.56 (.42 to .67)^a^	.23 (−.01 to .43)	.02 (−.03 to .12)	.11 (−.09 to .29)
Parietal Cortical Gray	.03 (−.07 to .13)	.68 (.56 to .76)^a^	.32^a^ (.10 to .51)	.07 (−.03 to .17)	.05 (−.13 to .23)
Occipital Cortical Gray	.02 (−.09 to .02)	.65 (.53 to .74)^a^	.52 (.31 to .67)^a^	.03 (−.08 to .07)	.20 (.02 to .36)^a^

Values given are *n* (95% confidence interval), estimated from the combined sample of MZ and DZ twins. The cross-member within-trait correlation for Sz (Sz_m1_-Sz_m2_) is constrained to be .92 in MZ pairs and .515 in DZ pairs; the threshold fixed to give a 1% prevalence. The within-member cross-trait correlations are constrained such that Sz_m1_-BV_m1_ = Sz_m2_-BV_m2_, and the cross-member cross-trait correlations are constrained such that Sz_m1_-BV_m2_ = Sz_m2_-BV_m1_. BV, brain volume; MRI, magnetic resonance imaging; subscript m, member of a pair; other abbreviations as in Table 1.

a Confidence intervals not including zero indicate significance.

**Table 3:** Standardized Estimates of the Full Bivariate ACE Genetic Models for Sz and Each MRI BV

	*h*^*2*^_*BV*_	*c*^*2*^_*BV*_	*e*^*2*^_*BV*_	*r*_*g*_	*r*_*c*_	*r*_*e*_	*r*_*ph*_
Cerebrum	.76 (.57/.82)^a^	.00 (0/.18)	.24 (.18/.32)^a^	−.21 (−.42/−.02)^a^	−.99 (−1/1)	−.37 (−.57/−.14)^a^	−.22 (−.30/−.14)^a^
Cerebral Gray	.34 (.00/.63)	.23 (0/.53)	.43 (.34/.55)^a^	−.33 (−.1/.16)	1 (−1/1)	−.36 (−.55/−.15)^a^	−.08 (−.16/.00)
Cerebral White	.63 (.32/.79)^a^	.10 (0/.38)	.27 (.21/.36)^a^	−.08 (−.41/.13)	−.97 (−1/1)	−.09 (−.31/.15)	−.17 (−.25/−.09)^a^
Lateral Ventricles	.71 (.31/.80)^a^	.02 (0/.39)	.27 (.20/.37)^a^	.11 (−.27/.47)	−.99 (−1/1)	.41 (.13/.65)^a^	.10 (0/.19)
3rd Ventricle	.64 (.29/.81)^a^	.11 (0/.40)	.25 (.18/.33)^a^	−.06 (−.21/.43)	1 (−1/1)	.23 (−.07/.49)	.19 (.09/.28)^a^
Frontal Cortical Gray	.43 (.00/.67)	.13 (0/.53)	.44 (.33/.57)^a^	.01 (−1/1)	.51 (−1/1)	−.54 (−.74/−.29)^a^	−.03 (−.13/.06)
Temporal Cortical Gray	.52 (.13/.65)^a^	.02 (0/.36)	.45 (.34/.59)^a^	−.04 (−.56/.35)	.99 (−1/1)	−.34 (−.58/−.07)^a^	−.04 (−.14/.06)
Parietal Cortical Gray	.67 (.29/.76)^a^	0 (0/.34)	.33 (.24/.44)^a^	.08 (−.27/.46)	.57 (−1/1)	−.26 (−.50/.02)	.03 (−.07/.13)
Occipital Cortical Gray	.21 (.00/.61)	.43 (.06/.64)^a^	.36 (.27/.48)^a^	−.48 (−1/.07)	1 (1/1)^a^	−.08 (−.33/.18)	.01 (−.09/.11)

Values given are *n* (95% confidence interval). *h*^*2*^*, c*^*2*^*, e*^*2*^ = standardized additive genetic, shared environmental, and nonshared environmental variance components; *r*_*g*_, *r*_*c*_, *r*_*e*_ = genetic, shared, and unshared environmental correlation; *r*_*ph* =_ total phenotypic correlation. Fixed (genetic) model for Sz used: *h*^2^=.81, *c*^2^=.11, *e*^2^=.08 and prevalence of 1%. Abbreviations as in Tables 1 and 2.

a Confidence intervals not including zero indicate significance.
