# Supplementary material for: The Genetic and Environmental Determinants of the Association Between Brain Abnormalities and Schizophrenia: The Schizophrenia Twins and Relatives Consortium
Source: Biol Psychiatry. 2012 May 15;71(10):915–21. doi: 10.1016/j.biopsych.2012.01.010 (PMC3343260; doi:10.1016/j.biopsych.2012.01.010)
Supplement: Supplement 1 [file mmc1.pdf]

The Genetic and Environmental Determinants of the Association Between Brain Abnormalities and Schizophrenia: The Schizophrenia Twins and Relatives Consortium

Supplemental Information

Recruitment

The Dutch sample contributed three cohorts. The discordant twin sample (MZ and DZ) have been described previously (1,2). The control twins (1,3,4) were recruited from the twin sample of the Department of Psychiatry at the University Medical Center Utrecht, the Netherlands and from the population based Netherlands Twin Register (5). Healthy control twins from the University Medical Center Utrecht bipolar twin study were also added (6). The Finnish twins were drawn from a twin cohort comprised of all of the same-sex twins born in Finland from 1940 through 1957 in which both members of each pair were alive and residing in Finland as of 1967 (7). In the United Kingdom, probands were referred to the study from across the country by their consulting psychiatrists. Control twins were recruited from the Institute of Psychiatry Volunteer Twin Register and by national media advertisements. The German twin sample was collected as part of the Heidelberg-Jena twin study on schizophrenia. Twins from all sites, except Finland, were recruited through advertisements and in collaboration with treating hospitals from the surrounding areas.

Assessment

All subjects underwent extensive psychiatric evaluation using available hospital records and structured psychiatric interviews (Utrecht: the Comprehensive Assessment of Symptoms and History interview (8), the Schedule for Affective Disorders and Schizophrenia: Lifetime Version (9), the Structured Interviews for DSM-IV (10), the Family Interview for Genetic Studies (11), and a medical history inventory; Helsinki: Structured Clinical Interview for DSM-III-R Disorders (SCID), Patient or Nonpatient Edition respectively (12) and Structured Clinical Interview for DSM-III-R Personality Disorders (13); London:

Schedule for Affective Disorders and Schizophrenia—Lifetime Version (9); Jena: Schedules for Clinical Assessment in Neuropsychiatry (14) and Family History Research Diagnostic Criteria (15)). At all sites psychiatric diagnosis was established according to DSM-IV criteria. The diagnostic interviews were conducted by trained and experienced psychologists and psychiatrists. In the case of doubt consensus was reached with a senior psychiatrist. For this study we excluded individuals with schizoaffective disorder. Exclusion criteria for all were the presence of significant medical or neurological illnesses including migraine, epilepsy, hypertension, cardiac disease, diabetes mellitus, endocrine disorders, cerebrovascular disease, alcohol or other drug dependence, significant past head trauma, or IQ below 80. Zygosity was based on DNA polymorphisms. Control twins were excluded in case of an axis I and/or schizophrenia-spectrum diagnosis.

Consent

The studies were approved by their respective ethics committees. All subjects gave written informed consent to participate in the study after a full explanation of the study aims and procedures.

Table S1. Scanner type and acquisition protocol used at each study site

Site	Scanner	Protocol/orientation	Voxel dimensions (mm)	TE (msec)	TR (msec)	Flip angle
Utrecht	Philips NT 1.5T	3D-FFE/coronal	1x1x1.2	4.6	30	30°
London Maudsley	GE Signa 1.5T	3D-SPGR/coronal	0.781x0.781x1.5	5	35	35°
London St Georges	GE Signa 1.5T	3D-SPGR/coronal	0.781x0.781x1.5	5	35	35°
Jena	Philips ACS II 1.5T	3D-FFE/sagittal	1x1x1	5	13	25°
Helsinki	Siemens Magnetom Impact 1.0T	MPRAGE/sagittal	1x1x1.2	4.4	11.4	12°

Table S2. Standardized estimates (with 95% confidence interval) of the bivariate AE genetic models for schizophrenia and relevant MRI brain volume.

	h^2_{BV}	e^2_{BV}	r_g	r_e	r_{ph}
Cerebrum	**.76** (.69 / .82)	**.24** (.18 / .31)	**-.21** (-.31 / -.10)	**-.37** (-.57 / -.14)	**-.22** (-.30 / -.14)
Cerebral white	**.73** (.65 / .80)	**.27** (.20 / .35)	**-.20** (-.31 / -.09)	-.07 (-.30 / .16)	**-.17** (-.25 / -.09)
Lateral ventricles	**.73** (.64 / .80)	**.27** (.20 / .36)	.05 (-.08 / .19)	**.42** (.14 / .65)	.10 (.00 / .20)
3rd ventricle	**.76** (.67 / .82)	**.25** (.18 / .33)	**.20** (.07 / .33)	**.22** (-.08 / .49)	**.18** (.08 / .28)
Temporal cortical gray	**.55** (.41 / .66)	**.45** (.34 / .59)	.03 (-.12 / .19)	**-.34** (-.58 / -.08)	-.04 (-.01 / .06)
Parietal cortical gray	**.68** (.57 / .76)	**.32** (.24 / .44)	.09 (-.05 / .23)	**-.25** (-.50 / .01)	.03 (-.07 / .13)

h^2, e^2 = standardized additive genetic and non-shared environmental variance components; r_g , r_e = genetic, and non-shared environmental correlation; r_{ph} = total phenotypic correlation. Confidence intervals not including zero indicate significance (bold). Fixed (genetic) model for schizophrenia used: h^2 = .81, c^2 = .11, e^2 = .08 and prevalence of 1%. A = additive genetic effects; E = unique environmental effects, including measurement error.

Supplemental References

1. Baare WF, van Oel CJ, Hulshoff Pol HE, Schnack HG, Durston S, Sitskoorn MM, Kahn RS (2001): Volumes of brain structures in twins discordant for schizophrenia. *Arch Gen Psychiatry* 58(1):33-40.

2. Hulshoff Pol HE, Brans RG, van Haren NE, Schnack HG, Langen M, Baare WF, *et al.* (2004): Gray and white matter volume abnormalities in monozygotic and same-gender dizygotic twins discordant for schizophrenia. *Biol Psychiatry* 55(2):126-130.

3. Posthuma D, De Geus EJ, Neale MC, Hulshoff Pol HE, Baare WEC, Kahn RS, Boomsma D (2000): Multivariate genetic analysis of brain structure in an extended twin design. *Behav Genet* 30(4):311-319.

4. Baare WF, Hulshoff Pol HE, Boomsma DI, Posthuma D, De Geus EJ, Schnack HG, *et al.* (2001): Quantitative genetic modeling of variation in human brain morphology. *Cereb Cortex* 11(9):816-824.

5. Boomsma DI (1998): Twin registers in Europe: an overview. *Twin Res* 1(1):34-51.

6. van der Schot AC, Vonk R, Brans RG, van Haren NE, Koolschijn PC, Nuboer V, *et al.* (2009): Influence of genes and environment on brain volumes in twin pairs concordant and discordant for bipolar disorder. *Arch Gen Psychiatry* 66(2):142-151.

7. Cannon TD, Kaprio J, Lonnqvist J, Huttunen M, Koskenvuo M (1998): The genetic epidemiology of schizophrenia in a Finnish twin cohort. A population-based modeling study. *Arch Gen Psychiatry* 55(1):67-74.

8. Andreasen NC, Flaum M, Arndt S (1992): The Comprehensive Assessment of Symptoms and History (CASH). An instrument for assessing diagnosis and psychopathology. *Arch Gen Psychiatry* 49(8):615-623.

9. Endicott J, Spitzer RL (1978): A diagnostic interview: the schedule for affective disorders and schizophrenia. *Arch Gen Psychiatry* 35(7):837-844.

10. American Psychiatric Association (1994): *Diagnostic and Statistical Manual of Mental Disorders. 4th edition (DSM-IV)*. Washington DC: American Psychiatric Association.

11. Nurnberger JI, Jr., Blehar MC, Kaufmann CA, York-Cooler C, Simpson SG, Harkavy-Friedman J, *et al.* (1994): Diagnostic interview for genetic studies. Rationale, unique features, and training. NIMH Genetics Initiative. *Arch Gen Psychiatry* 51(11):849-859.

12. Spitzer RL, Williams JB, Gibbon M, First MB (1989): *Instruction Manual for the Structured Clinical Interview for DSM-III-R (SCID).* New York, NY: Biometrics Research Department, New York State Psychiatric Institute.

13. Spitzer RL, Williams JB (1986): *Structured Clinical Interview for DSM-III-R Personality Disorders.* New York, NY: Biometrics Research Department, New York State Psychiatric Institute.

14. Wing JK, Babor T, Brugha T, Burke J, Cooper JE, Giel R, *et al.* (1990): SCAN. Schedules for Clinical Assessment in Neuropsychiatry. *Arch Gen Psychiatry* 47(6):589-593.

15. Andreasen NC, Endicott J, Spitzer RL, Winokur G (1977): The family history method using diagnostic criteria. Reliability and validity. *Arch Gen Psychiatry* 34(10):1229-1235.
